# Clear aligners for maxillary anterior en masse retraction: a 3D finite element study

**DOI:** 10.1038/s41598-020-67273-2

**Published:** 2020-06-23

**Authors:** Ting Jiang, Rui Ying Wu, Jian Kai Wang, Hong Hong Wang, Guo Hua Tang

**Affiliations:** 1 Department of Orthodontics, Shanghai Ninth People’s Hospital, College of Stomatology, Shanghai Jiao Tong University School of Medicine, Shanghai, 200011 China; 2 National Clinical Research Center for Oral Diseases; Shanghai Key Laboratory of Stomatology & Shanghai Research Institute of Stomatology, Shanghai, 200011 China

**Keywords:** Dental treatment planning, Cone-beam computed tomography

## Abstract

To evaluate tooth behaviours under various maxillary incisor retraction protocols for clear aligner therapy. A three-dimensional finite element model of maxillary dentition was constructed for first premolar extraction. A loading method was developed to mimic the mode of action of clear aligners for incisor en masse retraction. Three protocols with different amounts of retraction and intrusion on incisors were designed. Initial tooth displacements and stresses on periodontal ligaments were analysed with ANSYS software. The central (U_1_) and lateral (U_2_) incisors exhibited uncontrolled lingual tipping and extrusion upon 0.25 mm retraction. U1 exhibited translation movement, while U_2_ underwent less tipping during 0.2 mm retraction and 0.15 mm intrusion. Labial tipping and intrusion of U_1_ and bodily intrusion of U_2_ were observed during 0.1 mm of retraction and 0.23 mm of intrusion. With the additional intrusion on incisors, canine showed extrusion movement, and higher stresses on periodontal ligaments were shifted from U_2_ to canines. Incisors also exhibited different mesial-distal angulation in the three simulations, while posterior teeth all suffered mesial inclination. Incorporating intrusion displacement in clear aligners led to a tendency of lingual root movement during incisor retraction. The complexity of tooth movement should be recognized regarding clear aligner therapy.

## Introduction

In 1997, Align Technology (Align Technology, California, USA) introduced clear aligner treatment (CAT). In this system, serial thermoplastic aligners were produced based on the stages of tooth movement simulated in a computer. Each aligner is worn for 1 to 2 weeks to gradually move misaligned teeth to planned positions. As an inconspicuous alternative to traditional fixed instruments, CAT has attracted an increasing number of adult patients seeking orthodontic therapy^1^.

In the early stages, clear aligners were only considered to treat simple orthodontic problems of mild or moderate anterior crowding^2^. With the development of computer technology and the gradual recognition of the biomechanical properties of aligner materials, CAT has demonstrated the capacity to treat more complex cases, such as cases requiring tooth extraction^3–6^. Nevertheless, these case reports pointed out the limitations of using clear aligners to complete the gap closure in extraction treatment. Clear aligners are not rigid enough to retain their original shape in space closure, which might result in torque loss and adverse extrusion of the anterior teeth^7–10^. Therefore, a certain amount of intrusion is intentionally added during the setup when the incisors are designed to be retracted. However, the effectiveness of extra intrusion in controlling the movement of incisors has not been proven, and the most efficient ratio between the amount of retraction and intrusion is unclear.

Finite element analysis can calculate initial tooth movement instantly after force loading. It has been widely used in biomechanics to analyse the stress and strain response of external forces in residential structures and has been demonstrated to be an effective tool to simulate tooth displacement patterns in orthodontics^11,12^.

The purpose of this study is to determine the behaviours and stress distributions of both anterior and posterior teeth under different amounts of retraction and intrusion protocols with clear aligners by three-dimensional finite element (3D FE) analysis.

## Methods

**Figure 1:**
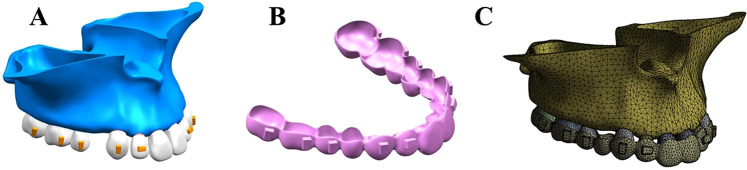
Three-dimensional finite element model for incisor retraction in a 1^st^ premolar extraction case. (**A**) Maxillary arch with 1^st^ premolar removal and attachments on the vestibular surfaces of crowns. (**B**) Geometric model of the clear aligner. (**C)** Mesh division on the model assembly with the aligner on dentition.

3D FE models of the maxillary dentition with extracted first premolars, periodontal ligaments (PDLs), and alveolar bone were built using GEOMAGIC studio (Raindrop Geomagic, North Carolina, USA). Teeth and maxillary bone were reconstructed based on cone-beam computed tomography (CBCT) scanning of an adult male subject with well-aligned dentition and a normal upper incisor to maxillary plane of 110 degrees^13^. PDLs were fabricated as a linear elastic film with an average thickness of 0.25 mm around the roots of all teeth^14^. To simulate a sequential pattern of space closure by which canines were distalized first followed by en masse retraction of incisors, a 0.5 mm space was designed in the model between canines and lateral incisors^15^. Horizontal rectangular attachments (2 mm height, 3 mm width, and 1 mm thickness) were designed for lateral incisors, and vertical rectangular attachments (3 mm height, 2 mm width, and 1 mm thickness) were designed for teeth other than centre incisors. The aligners had a thickness of 0.38 mm, in which external offset devices for all crowns and attachments were developed in the simulation (Fig. 1A,B)^16^. All components were imported for FE analysis by ANSYS Workbench 15.0 (Ansys, Pennsylvania, USA).

### Material properties

Teeth and attachments were regarded as isotropic and homogeneous materials with rigid stiffness properties, while PDLs were set up as hyperelastic materials to mimic its true mechanical properties as much as possible. In the calculation of the stress on the PDLs, the alveolar bone was designed as a rigid body. Aligners were set as isotropic and homogeneous materials (Table 1).

**Table 1:** Material properties used in FE model.

Material	Young’s modulus (MPa)	Poisson’s ratio
Tooth	1.96 * 10^4^	0.30
Periodontal ligament	0.69	0.45
Alveolar bone	1.37 * 10^3^	0.30
Attachments	12.5 * 10^3^	0.36
Clear aligner	528	0.36

The FE mesh was divided by the discretization process, and 234,691 nodes and 13,101 linear elements were generated (Fig. 1C). A friction condition was established in contact interfaces between the aligner and tooth crown surface and attachments, with a friction coefficient of μ = 0.2^17^.

### Establishment of a coordinate system

The mesial incisal points of the central incisors and mesial cusp tips of the bilateral first molars were used to define the occlusal plane^18^. The X-axis represents the direction of the coronal plane with the positive direction being towards the mesial surface of the tooth; the Y-axis represents the sagittal plane with the positive direction being towards the lingual surface; and the Z-axis represents the vertical plane with the positive direction being towards the gingival tissue.

### Configuration setting

**Figure 2:**
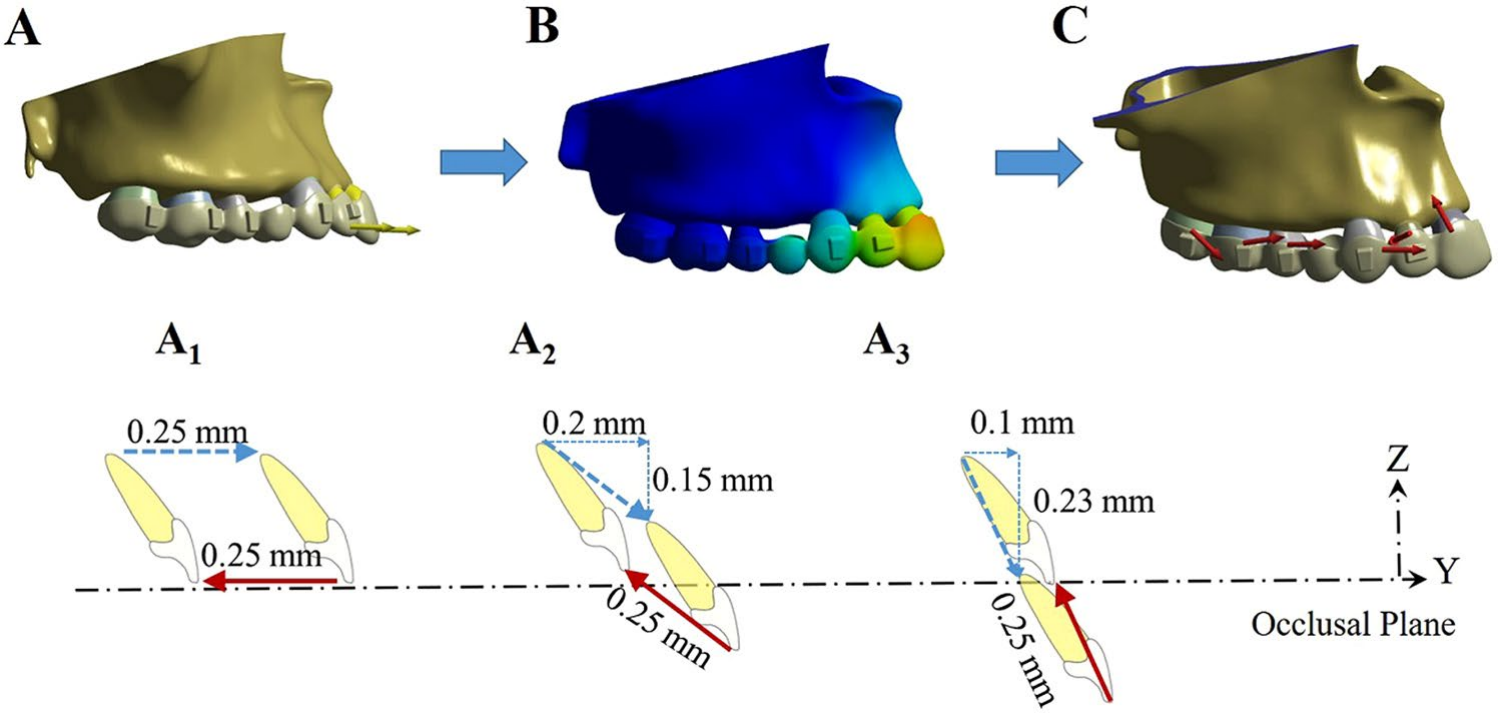
Loading method of the finite element model to simulate incisor retraction with a clear aligner. The incisors were first displaced in the opposite direction of retraction (**A**). This led to the deformation of the aligner matching the dentition (**B**). The forces generated by the aligner on each tooth were then calculated and finally loaded back on the corresponding tooth in the reverse direction (**C**). Three retraction protocols were designed with different amounts of retraction and intrusion with the same total amount of movement of 0.25 mm (A_1_, A_2_ and A_3_). Dashed arrows indicate the vectors of incisor displacement that activate the aligner. Solid arrows indicate the vectors of the designed incisor retraction.

Three retraction protocols were proposed (Fig. 2):

- Configuration 1 (A_1_): retraction of incisors bodily by 0.25 mm along the occlusal plane,
- Configuration 2 (A_2_): 0.2 mm retraction with 0.15 mm intrusion, and
- Configuration 3 (A_3_): 0.1 mm retraction with 0.23 mm intrusion.

For all three protocols, the distance of movement measured on the tip of the root was 0.25 mm, as recommended by Invisalign^19,20^.

### Loading method

To mimic the action of the aligner on the tooth in the FE model, a new loading method was developed (Fig. 2). In configuration 1, for example, the incisors were intended to be retracted by 0.25 mm (Fig. 2A_1_). The incisors were first translated labially along the occlusal plane for 0.25 mm (Fig. 2A). This led to the deformation of the aligner fitting on the dentition (Fig. 2B). The forces generated by the aligner on each tooth were then calculated by the software and then loaded back on the corresponding tooth in the reverse direction (Fig. 2C).

Informed consent for study participation was obtained, and the use of the CBCT data was approved by the Ethics Committee of Shanghai Ninth People’s Hospital (SH9H-2018-T63-1). All experiments were approved and performed in accordance with relevant guidelines and regulations.

## Results

**Figure 3:**
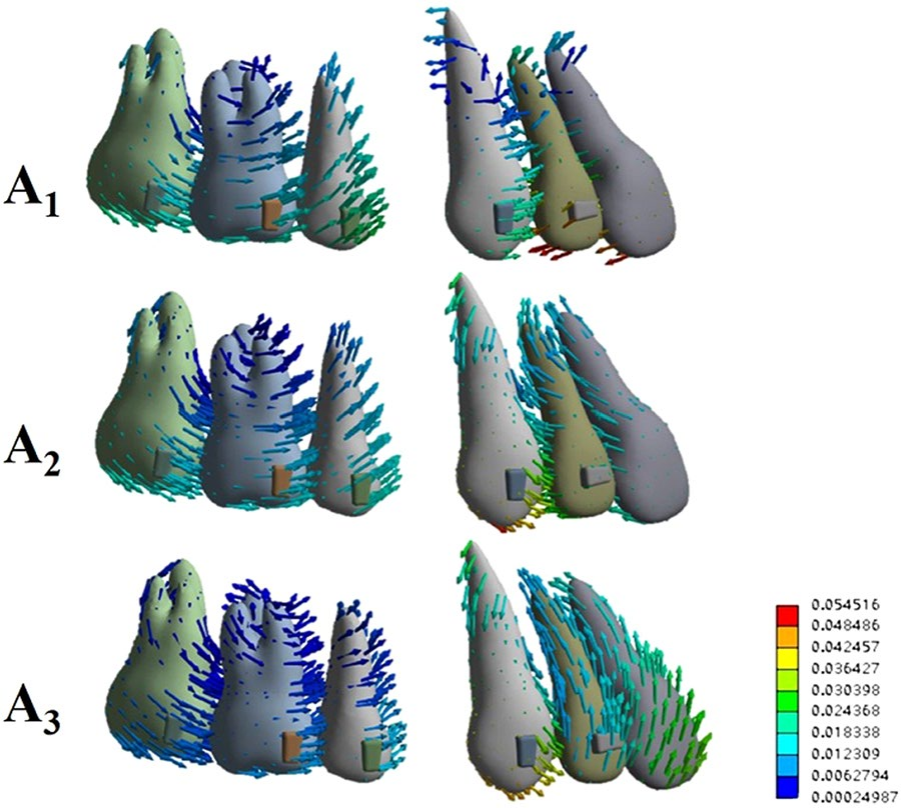
Vector analysis of the overall displacement pattern of the dentition for the three incisor retraction protocols: A_1_, A_2_ and A_3_.

**Figure 4:**
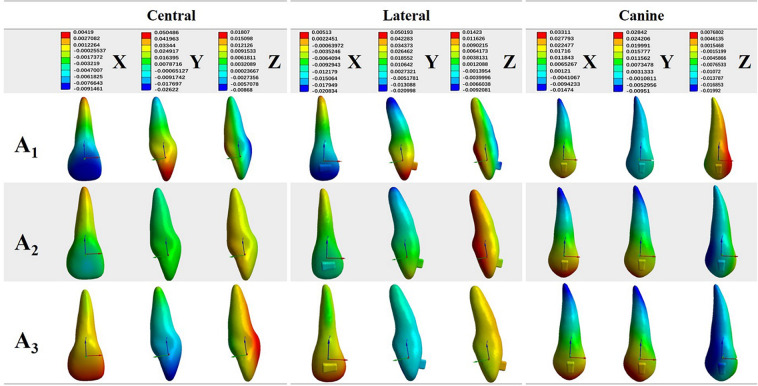
The displacement pattern of incisors and canines in each direction for the three incisor retraction protocols: A_1_, A_2_ and A_3_.

**Figure 5:**
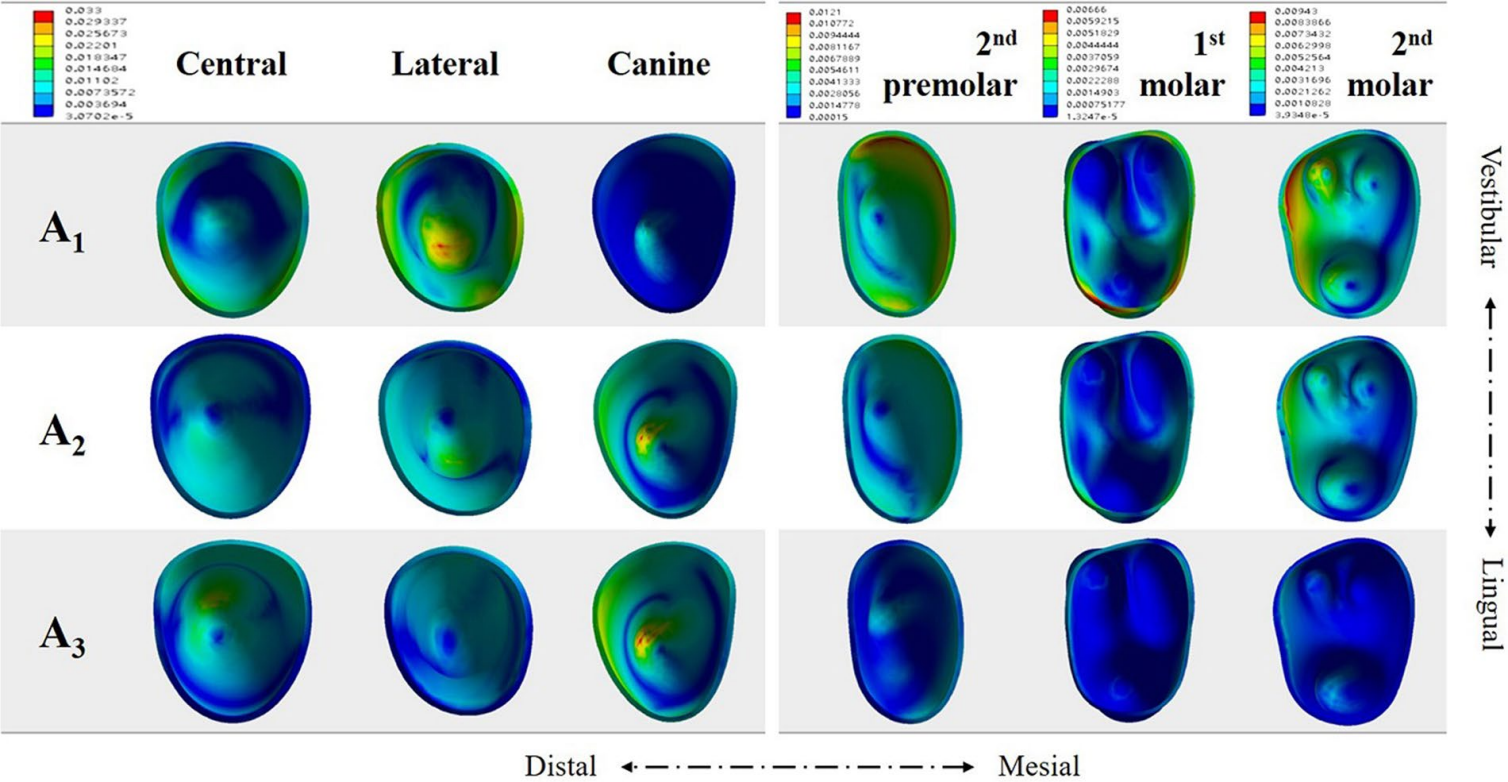
Stress distribution around the periodontal ligament of each tooth for the three incisor retraction protocols: A_1_, A_2_ and A_3_.

During incisor en masse retraction, different force systems and behaviours of both anterior and posterior teeth were recorded among the three performed simulations (Figs. 3–5). To facilitate the comparison, the coordinate values under different simulations were unified for each tooth type.

### A_1_: 0.25 mm retraction

Upon this retraction protocol, both central and lateral incisors experienced maximum lingual displacement on incisal edges, while the root apex performed a movement in the labial direction, resulting in uncontrolled lingual tipping movement. In contrast, the canine and posterior teeth exhibited mesial inclination (Fig. 3A_1_).

Detailed analysis of anterior tooth displacement showed that both central and lateral incisors had distal crown and mesial root tipping (Fig. 4A_1_, X-axis). The lingual tipping movement (Y-axis) was accompanied by extrusion on the labial surfaces (Z-axis). Canines exhibited mesial crown tipping, intrusion and minor mesial lingual rotation.

The maximum stress on the PDL was located at the root apex of the lateral incisor, followed by the cervical area of the lateral and central incisors (Fig. 5A_1_). For posterior teeth, the mesial cervical area of the 2^nd^ premolar and distal cervical area of the 2^nd^ molar also showed higher stress.

### A_2_: 0.2 mm retraction and 0.15 mm intrusion

The displacements in the crown and root of the central incisor were relatively uniform, indicating bodily movement (Figs. 3A_2_ and 4A_2_). The lateral incisors still underwent a lingual tipping movement, albeit less apparent than that in A_1_. While both incisors showed intrusion movement, more intrusion was noted on lateral incisors. Compared to A_1_, both incisors displayed less distal crown angulation (X-axis). Canine and posterior teeth also exhibited mesial tipping movements. However, lingual crown tipping and extrusion were noted on canines. The stress on the PDL was now concentrated at the apical area of the canine (Fig. 5A_2_). The amount of stress on the posterior teeth was decreased when compared to that in A_1_.

### A_3_: 0.1 mm retraction and 0.23 mm intrusion

The maximum displacement of the central incisor was observed on the crown, showing a labial crown tipping movement, and the intrusion was significant (Figs. 3A_3_ and 4A_3_). The movement pattern of the lateral incisor resembled bodily intrusion with minor labial crown tipping. Unlike in A_1_ or A_2_, in A_3_, the incisors exhibited mesial crown tipping on the X-axis (Fig. 4A_3_). Canines exhibited very similar displacement to that observed in A_2_, with mesial crown tipping, extrusion and slightly more lingual crown tipping. The stresses on the PDL were also concentrated at the apical area of the canine, while the stresses were smaller and more uniform on incisors and posterior teeth (Fig. 5A_3_).

**Figure 6:**
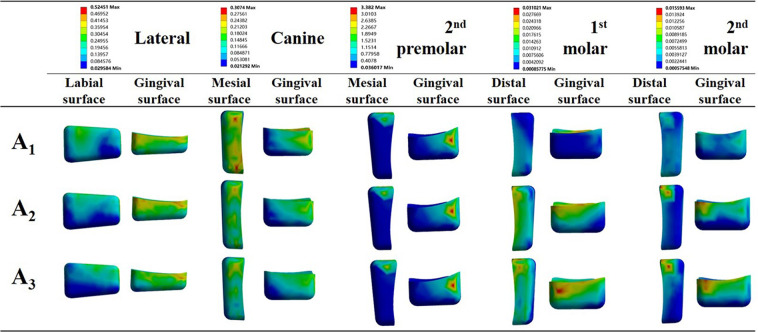
Stress distribution around the attachments for the three incisor retraction protocols: A_1_, A_2_ and A_3_. Surfaces with higher stresses are illustrated.

The stress distributions on attachments were similar among the three simulations. Higher stresses were concentrated on the gingival surfaces of the lateral incisor and 2^nd^ premolar, mesial surfaces of canines, and distal surfaces of molars (Fig. 6). The maximum stresses were noted on the 2^nd^ premolars for all the simulations. Much lower stresses were observed on the 1^st^ molar attachments, and the lowest were recorded on the 2^nd^ molars. With the increase in intrusion on incisors, the stresses on the gingival surfaces of the attachments on the 1^st^ and 2^nd^ molars increased.

## Discussion

Despite the growing consumer demand and worldwide use of clear aligners to treat misaligned teeth, several concerns regarding the efficacy of the system in controlling tooth movement remain. The tooth movement achieved upon aligner therapy differs from the tooth movement planned by the virtual setup^21^. This less predictable movement might be due to the fact that the force-transmission mechanisms of clear aligners are not yet well clarified. In fixed-appliance treatment, the force originating from metal wire and bracket interactions is transmitted to a certain point on the tooth, causing displacement. In contrast, clear aligner therapy outcomes are the result of a predetermined mismatch between the tooth and aligner. The force application point is ever changing, and the direction and amount of the force are very elusive.

Several experimental approaches have been carried out on the biomechanics of possible tooth movement with clear aligners. Compared to displacement measurements and strain gauge measurements, 3D FE analysis has the advantage of taking periodontal tissues into consideration and can calculate the stress, strain and displacement of any part of the tooth and aligner^11,22–27^. However, most FE studies on clear aligners were limited to a single tooth or a segment of teeth^11,22–24,26^. In addition, the loading method was conventionally described as moving the assembled aligner directly, which was inconsistent with clinical reality^26^. In this study, the incisors were first forced in the reverse direction of retraction to activate the aligner. The forces resulting from the deformation of the aligner were then calculated by FE analysis, which were in turn loaded back on the corresponding teeth. The contact interfaces between the aligner and the crown surface and attachments were set to a friction coefficient of 0.2, thus mimicking the mode of action of a shape-mismatched aligner on the dentition. To our knowledge, this is the first time that a clear aligner FE model with intact maxillary dentition and attachments was constructed, and different protocols of tooth movement were analysed. The results showed that the method was practical and reliable.

Controlling the movement of incisors at will is a challenge for extraction treatment. In most cases, translation or controlled tipping is preferred for the retraction of upper incisors. In fixed appliances, the moment/force ratios at the bracket are the primary determinant of controlling the tooth movement^28,29^. It can also be achieved by positioning mini-screws and hooks to adjust the “line of force” related to the centre of resistance of the anterior segment in sliding mechanics^30,31^. However, the biomechanics of en masse retraction of incisors with clear aligners are not well understood.

Previous studies have reported that clear aligners caused tooth movement mainly by tilting motions and that bodily movement for extraction space closure was less successful^1,21^. Our present study showed that incisors exhibited uncontrolled tipping and undesired extrusion when bodily retraction was designed (Fig. 3). Thus, similar to fixed appliances, a “bowing effect” would occur during incisor retraction with clear aligners. This might be caused by forces acting below the resistance centres of the four incisors, and the aligner was not rigid enough to hold the teeth vertically. It is thus logical to allow for a certain amount of intrusion during the retraction movement. According to Invisalign, tooth movement is limited within 0.25 mm per stage^19,20^. Therefore, we designed two retraction and intrusion combinations for incisors, and the total movement amount was 0.25 mm (Fig. 2). On the basis of our FE results, intrusion movement helped generate a force system that approximated the bodily movement of incisors. As the amount of intrusion movement increased, the root movement in the lingual direction became more prominent. However, this tendency declined from the centre to the lateral sides, which produced inconsistent types of movement between the central and lateral incisors. Apart from their different positions in the dental arch, the discrepancy between central and lateral incisors might result from the different anatomical configurations of the crowns.

Anchorage is another major concern during space closing in extraction cases. To maximize the anchorage value of the posterior segment, a protocol of sequential retraction of the canines and incisors was recommended^15^. Our present study adopted this concept so that 0.5 mm space was created mesial to the canines on the FE model to mimic canine distalization. This gap also increased the aligner surface area around the canine and incisor crowns. Meanwhile, the flexibility of the edentulous span of the aligner distal to the incisor segment was effectively reduced by canine positioning^15^. In our results, the posterior teeth and canines showed similar mesial tipping movement in the three protocols. With the decrease in the amount of retraction in A_2_ and A_3_, less mesial migration of the posterior teeth was observed. However, canines revealed more extrusion displacement when more intrusion was planned on the incisors (Figs. 3 and 4). Higher stresses on canine PDLs were detected (Fig. 5). Based on these results, intraoral elastics could be placed on canines to reinforce anchorage. Taking the vertical movement of canines into consideration, class II elastics should be used for protocol A_1,_ and elastics supported by mini-screws on posterior maxillary bone would be beneficial for protocols A_2_ and A_3_.

Auxiliaries such as attachments were mandatory in clear aligner therapy to achieve desired results^25,26,32^. While some reports pointed out that customized attachments could facilitate complex movements, others failed to find significant differences in the shape and position of attachments on tooth movement^16,20,22,26^. Most likely, the effect of the attachment would depend on the shape of the tooth. In our present study, only conventional rectangular attachments were used for convenience. Much higher stresses on the PDLs and gingival surfaces of the attachments were detected on lateral incisors (Figs. 5 and 6). These results were in agreement with previous findings that upper lateral incisors commonly come off track during space closure, and horizontal bevelled attachments are recommended for better retention^33^. Higher stresses were also observed on the gingival surfaces of the attachments on the 1^st^ and 2^nd^ molars when more intrusion was placed on incisors (Fig. 6). This emphasized the significance of the attachments on molars to support incisor intrusion.

Considering all the results obtained through the finite element analysis, it could be stated that the incisors underwent a transition from uncontrolled tipping to gradual root-controlled movement after intrusion displacements were planned. It is worth noting that intrusion was always accompanied by various movement types. Thus, assessment before treatment was indispensable from a clinical perspective. For patients with a deep overbite, more intrusion could not only contribute to improving the occlusion but also lead to good control of root lingual movements. Moreover, the different movement types on incisors draw attention to the clinical aspects of biomechanical analysis, showing that different forces might be loaded on incisors, which should be considered by orthodontists when planning clear aligner therapy.

Our present results were based on the fact that the upper incisors were on normal labiolingual inclination. Calibrating the amount of intrusion and retraction movement in detail according to different inclinations of incisors would help clinicians with treatment planning for individual cases. Although the reliability of finite element analysis must be verified, further studies examining different thicknesses and configurations of the aligners, as well as various attachment designs, should be carried out for a better understanding of clear aligner treatment.

## Conclusions

In this study, a unique loading method was developed to mimic the mode of action of clear aligners for anterior en masse retraction in a 3D FE model. The results showed that incorporating intrusion displacement on aligners led to a tendency of lingual root movement for incisor retraction. The discrepancy between the tooth movement displayed in the FE model and the tooth movement planned for incisors reiterated the complexity of the force system in clear aligner therapy.
